# Onychomycosis with gray-green staining caused by *Fusariumsolani*

**DOI:** 10.1016/j.mmcr.2024.100684

**Published:** 2024-11-16

**Authors:** Yoshihito Mima, Masako Yamamoto, Koichi Makimura, Ken Iozumi

**Affiliations:** a Department of Dermatology, Tokyo Metropolitan Police Hospital, Tokyo, Japan; b Teikyo University Institute of Medical Mycology, Tokyo, Japan; c Department of Dermatology, Misato Kenwa Hospital, Saitama, Japan

**Keywords:** Fusarium solani, Onychomycosis, *Pseudomonas aeruginosa*, Pigment, Dark green colony

## Abstract

We report a case of onychomycosis due to Fusarium solani with gray-green staining, which improved after nail plate removal and antifungal liquid of effinaconazole. Fungal cultures revealed light-brown and dark-green colonies. Gray-green nail might have occurred due to the combination of these colonies, which necessitated differentiation from green nail. Fusarium solani was detected on the genetic analysis of the colonies. Fusarium species reportedly produce yellow or red pigments; however, Fusarium species has not been previously reported to produce green pigments or forming dark-green colonies in fungal cultures.

## Highlights

• Only *F. solani* was detected, and repeated bacterial cultures were negative.
• There have been no reports of *Fusarium* species producing green pigments.
• Nail plate removal and antifungal ointment successfully treated the infection.

## 1 Introduction

*Fusarium* species are non-dermatophytic molds commonly found as soil saprophytes and are significant plant pathogens associated with various diseases such as crown rot, head blight, and scab on cereal grains [1]. *Fusarium* species can infect both humans and animals [2,3] and cause various types of infections, including superficial infections such as onychomycosis, locally invasive infections such as fungal abscess, or disseminated infections such as fungemia and organ infiltration [3]. Individuals with prolonged neutropenia and T-cell immunodeficiency are at higher risk for severe *Fusarium* species infections [4]. As a result, immunocompromised patients such as hematopoietic stem cell transplant recipients with severe graft-versus-host disease are more susceptible to invasive and disseminated *Fusarium* species infections [4].

*Fusarium* species such as *Fusarium oxysporum*, *Fusarium solani*, *Fusarium fujikuroi*, and *Fusarium lactis* reportedly cause onychomycosis [5,6]. A review on 86 cases of onychomycosis caused by *Fusarium* species reported that *F*. *solani* had the highest proportion, accounting for 44.11 % of the causative fungi, followed by *F. fujikuroi*, which accounted for 17.64 % of cases [6].

The clinical skin manifestations of onychomycosis caused by *Fusarium* are similar to nail infections caused by other fungi and include distal lateral and proximal subungual onychomycosis, total dystrophic onychomycosis, and superficial white onychomycosis [6]. Nails affected by *Fusarium* species may appear white or yellow, with nail plate thickening, cloudiness, and hyperkeratosis [6,7]. Herein, we present a unique case of a gray-green colored nail due to *F. solani*, which formed a dark-green colony in a fungal culture.

## 2 Case

**Fig. 1:**
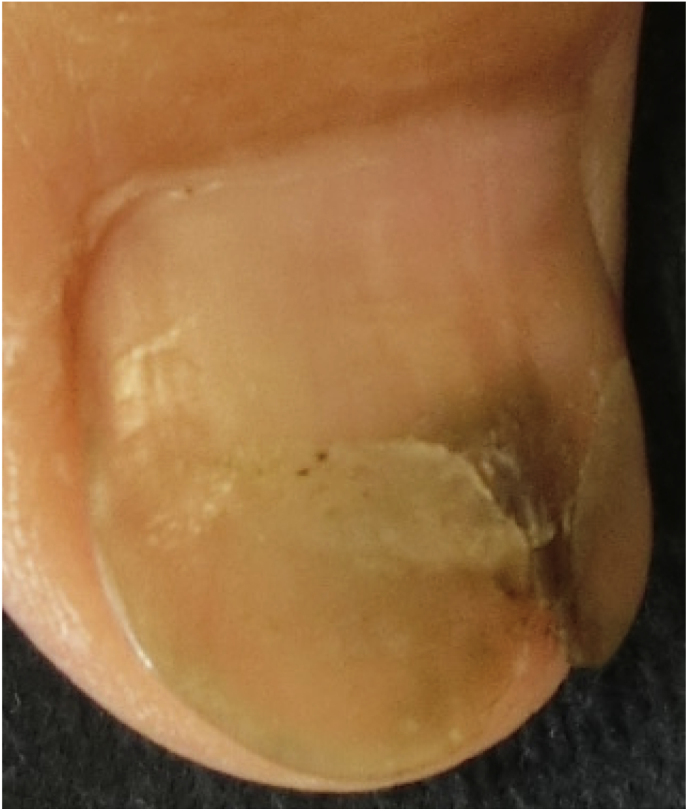
Clinical feature: the patient's thumbnail had a gray-green area. (For interpretation of the references to color in this figure legend, the reader is referred to the Web version of this article.)

A 76-year-old Japanese male without any significant past medical history presented with a gray-green colored thumbnail (Fig. 1, day 0). He had repeated episodes of nail wounds when he grew vegetables at home. Therefore, the possibility of a green nail (GN) due to *Pseudomonas aeruginosa* infection from a wound was suspected.

**Fig. 2:**
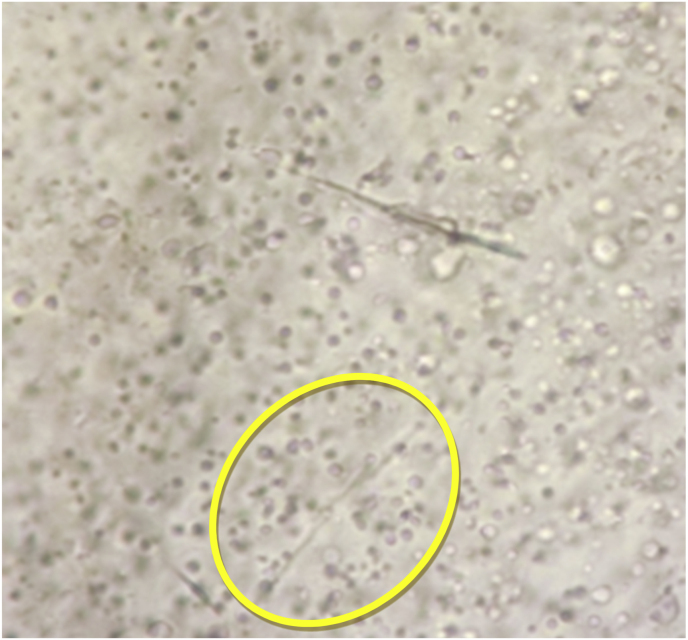
Microscopic feature (x200): the fungal test of the green nail revealed multiple spores (yellow circle). (For interpretation of the references to color in this figure legend, the reader is referred to the Web version of this article.)

**Fig. 3:**
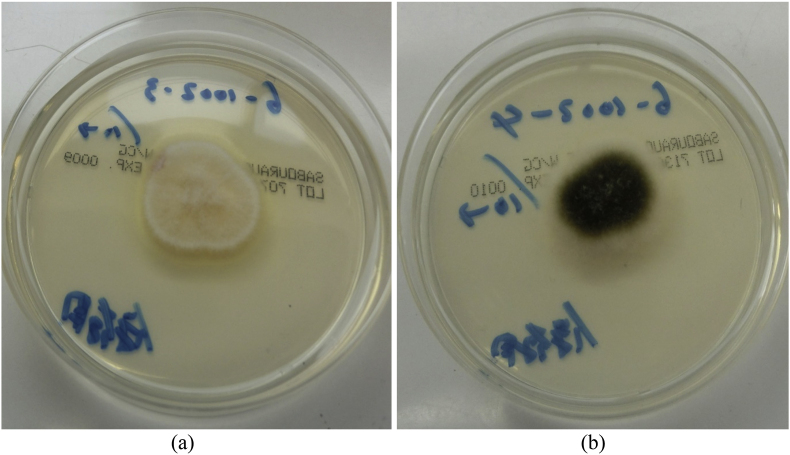
Clinical feature: two fungal colonies [a light brown colony (a) and a dark green colony (b)], were detected on subsequent tests. (For interpretation of the references to color in this figure legend, the reader is referred to the Web version of this article.)

**Fig. 4:**
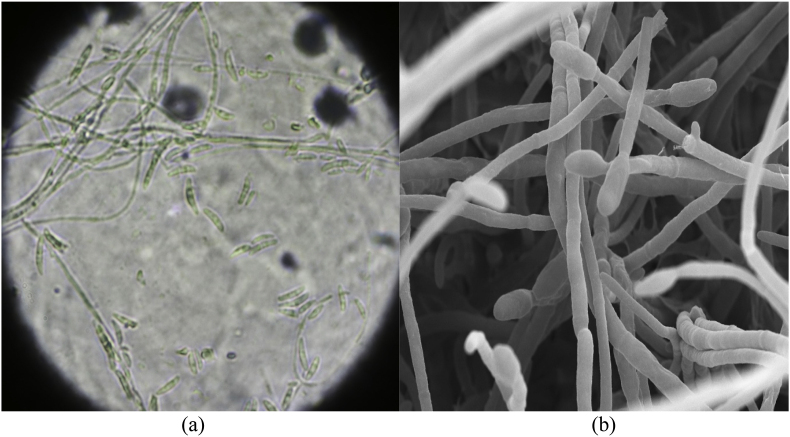
Microscopic feature: microscopy findings (a: x100) and electron microscopy examination (b: x400) revealed multiple microconidia and long, slender monophialides.

**Fig. 5:**
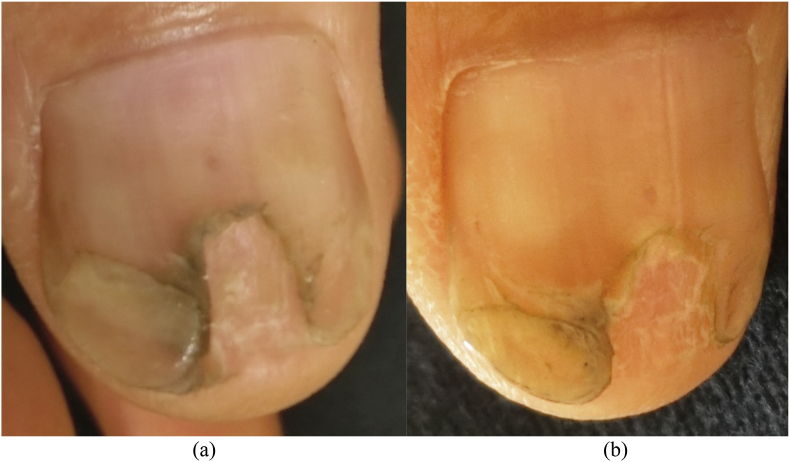
Clinical feature: after the onycholytic lesion was opened, effinaconazole ointment administration resulted in clinical improvement at 2 weeks (a) and 4 weeks (b).

No bacteria were detected on an initial bacterial culture (day 0). Given the possibility of a false negative result for *P. aeruginosa* [8], topical 1 % nadifloxacin was administered (day 14), but no improvement was observed after 5 months (day 151), and repeated bacterial cultures continued to yield negative results (day 0–151). GN can reportedly occur due to mixed infections of *P*. *aeruginosa* and fungi [9]. Considering the possibility of fungal infection, a fungal microscopic test of the nail was performed (day 151), which was positive (Fig. 2), and the patient was diagnosed with onychomycosis. Nail samples were cultured on Sabouraud Dextrose Agar, and light brown (strain #6-1002-3; Fig. 3a) and dark green (strain #6-1002-4; Fig. 3b) colonies were detected on the fungal culture. Microscopic examination of the dark green colonies revealed multiple microconidia and long, slender monophialides (Fig. 4a), which were confirmed using electron microscopy (Fig. 4b). After the two colonies were subcultured into potato dextrose agar medium, genetic analyses of these colonies, including internal transcribed spacer ribosomal deoxyribonucleic acid, sterol C-14 reductase gene erg-3 and translation elongation factor 1-a, were performed. The genetic analysis results of the light brown colony (strain #6-1002-3) and the dark green colony (strain #6-1002-4) are presented in Table 1, Table 2, respectively, both identifying the *F*. *(Neocosmospora) solani* complex. The patient was administered 10 % effinaconazole liquid (day 165), but the gray-green colored nail did not resolve after 1 month of treatment (day 193). The bacterial and fungal cultures were repeated, and only *F. solani* complex species were identified again. The onycholytic lesion was opened, and 10 % effinaconazole treatment was clinically effective at 2 (Fig. 5a) (day 207) and 4 (Fig. 5b) (day 221) weeks, resulting in complete improvement (day 270). Considering the negative results of bacterial cultures, the genetic analysis results, and the clinical course, the patient was finally diagnosed as having onychomycosis with gray-green staining due to *F*. *solani*.

**Table 1:** Results of the genetic analysis using molecular biological techniques on the target genes of internal transcribed spacer ribosomal deoxyribonucleic acid (ITSrDNA), 28S ribosomal ribonucleic acid (28SrRNA), sterol C-14 reductase gene erg-3 (ERG) and translation elongation factor 1-α (EF1-a) from the light brown colored colony (strain#6-1002-3).

Strain#6-1002-3	Target gene	Identified species	Homology	Accession No.
**NCBI**	ITSrDNA	*Fusarium oxsporum* f sp*. conglatinans*	551/551(100 %)	DQ452447
		*Fusarium solani*	550/551(99.8 %)	MG561938
**Mycobank**		*Fusarium solani*	551/551(100 %)	DQ452447
**NCBI**	28SrRNA	*Fusarium solani*	551/551(100 %)	LT746274
**Mycobank**		*Fusarium oxsporum* f sp. *conglatinans*	551/551(100 %)	DQ452447
		*Fusarium solani*	546/546	AB498917
**NCBI**	ERG	*Fusarium solani* species complex	570/571(99.8 %)	DQ237188
**NCBI**	EF1-α	*Fusarium solani* species complex	744/746(99.7 %)	KF939495

**Table 2:** The result of the genetic analysis using molecular biological techniques on the target genes of internal transcribed spacer ribosomal deoxyribonucleic acid (ITSrDNA), 28S ribosomal ribonucleic acid (28SrRNA), sterol C-14 reductase gene erg-3 (ERG) and translation elongation factor 1-α (EF1-a) from the dark green colored colony (strain#6-1002-4).

Strain#6-1002-4	Target gene	Identified species	Homology	Accession No.
**NCBI**	ITSrDNA	*Fusarium solani* species complex	539/540(99.8 %)	MG561938
**Mycobank**		*Neocosmospora rubicola*	539/540(99.8 %)	KM231800
		*Fusarium solani* species complex	539/540(99.8 %)	JX524022
**NCBI**	28SrRNA	*Fusarium solani* species complex	539/540(99.8 %)	MG561938
**NCBI**	ERG	*Fusarium solani* species complex	570/571(99.8 %)	DQ237188
**NCBI**	EF1-α	*Fusarium solani* species complex	734/738(99.5 %)	KT313615

## 3 Discussion

*Fusarium* species are non-dermatophytic molds commonly found as soil saprophytes and significant plant pathogens [1]. In our case, the patient had a history of home gardening, suggesting that *F. solani* infection was possibly acquired from soil during vegetable cultivation. *Fusarium* species are prone to develop into invasive or disseminated infections, particularly in patients with neutropenia [4]. Although *Fusarium* infections are typically localized to the skin and nails, localized *Fusarium* infections are considered potential precursors to systemic *Fusarium* infection [4,10]. Thus, early detection and treatment of *Fusarium* infection is essential, even for localized *Fusarium* infections in the skin or nails.

A nail with green appearance was clinically diagnosed as GN, which is commonly caused by *P. aeruginosa* infection [11]. In cases of GN, bacteria are often undetectable in bacterial cultures, with approximately 65 % of cases showing no bacterial growth [8]. However, since topical antibiotics are effective in most cases, it is believed that false negatives are common [8]. However, in our case, only *F. solani* was detected, and repeated bacterial cultures were negative, without any response to topical 1 % nadifloxacin. Therefore, GN due to *P. aeruginosa* infection was initially considered because of the history of nail trauma and the gray-green discoloration; however, the nail condition was ultimately diagnosed as onychomycosis caused by *F. solani*.

The treatment with antifungal liquid of effinaconazole alone was not effective; however, when combined with nail plate removal, the medication was able to penetrate under the nail plate, possibly leading to an improvement in the nail discoloration.

In the present case, fungal cultures revealed light brown (strain #6-1002-3) and dark green (strain #6-1002-4) colonies. The nail appeared gray-green, likely due to the combination of colonies with these two colors, which initially necessitated differentiation from GN. Fungi such as *Fusarium* and *Aspergillus* species produce secondary metabolites that display pigments in various colors, including yellow, orange, red, green, purple, brown, and blue [12]. *Fusarium* species are known to produce yellow and red pigments such as azaphilones, naphthoquinones, and hydroxyanthraquinones [13], which may explain the development of the light brown colony (strain #6-1002-3). The soft rot fungus *Chlorociboria aeruginascens* produces the blue-green pigment xylindein [14]. Additionally, *Candida stellatoidea* and *Candida albicans* are known to produce metabolic byproducts that result in green pigments, forming green colonies [15]. There has been a report of GN caused by *Candida* species infection alone [16], which may be related to the production of green pigment metabolites by *Candida* species. While several fungi can produce green pigments [14,15], there have been no reports of *Fusarium* species producing green pigments or forming dark green colonies in fungal cultures, as seen in the present case. Further accumulation of cases and research are needed to understand the mechanism behind this green discoloration.

## CRediT authorship contribution statement

**Yoshihito Mima:** Writing – review & editing, Writing – original draft, Project administration, Data curation, Conceptualization. **Masako Yamamoto:** Writing – review & editing, Data curation. **Koichi Makimura:** Writing – review & editing, Data curation. **Ken Iozumi:** Writing – review & editing, Writing – original draft, Project administration, Data curation, Conceptualization.

## Ethical form

We declare no funding or potential conflicts of interests.

We obtained written consent form to publish the case report from the patient.

## Declaration of competing interest

There are none.
